# The Ice-Bag in Pneumonia

**Published:** 1897-03

**Authors:** 


### THE ICE-BAG IN PNEUMONIA.

With regard to pneumonia, Dr. Lees had, by request, given the speaker the notes and chart of two cases, in which, by early application of ice, the disease had apparently been cut short. Instead of a sustained high temperature for a week, and a sudden critical fall to 95° or 96° F. (35° or 35.6° C.), temperature had fallen steadily from the moment when the ice-bag was applied. Dr. Lees gives the following directions for use of the ice-bag in pneumonia:

1. Apply the ice-bag over the dull area, and especially over the advancing edge of the consolidation.
2. If the area is large, use two bags at least, or three. Even young children may require two.
3. Expect to find a distinct local effect (improvement of percussion-note, less bronchial breathing, looser rales) on careful physical examination, after the ice has been applied for twenty-four hours.
4. If fresh areas of consolidation develop, use additional bags. Four or even more may be needed in a bad case. The amount of the dose is as important as it is with drugs.
5. Take the temperature every half-hour for the first three hours; afterward, every two hours.
6. Apply hot-water bottles to the feet and legs. For children supply these before the ice is applied.
7. Examine the physical signs carefully twice daily and shift the bags accordingly.
8. If pericarditis is present place one ice-bag over the heart.
9. If temperature is below 99° F. (37.2° C.), or hands cold, or lips bluish, remove the bags for an hour; then replace them and use them for two or three hour periods, with one or two hour intervals.
10. If in a severe case there is distinct cyanosis and a rapid, feeble pulse, consider whether leeches (in urgent cases venesection) would not relieve the right heart.
11. In all cases see that sleep is secured during the first three or four nights. If the relief afforded by the ice-bags does not suffice for this, give chloralamid or morphine.

Pneumonia treated vigorously with ice within twenty-four hours after the rigor may sometimes be aborted.—*Universal Medical Journal.*

